# Gastro-intestinal stromal tumor (GIST) complicating a colonic interposition: a novel case report

**DOI:** 10.1186/1756-0500-7-604

**Published:** 2014-09-04

**Authors:** Maaz B Badshah, Haris Riaz, Mark A Korsten, Atiya Dhala, Yeun-Hee A Park, Maria Abadi, Mashood B Badshah

**Affiliations:** James J. Peters VA Medical Center/Mount Sinai School of Medicine, New York, NY USA; Department of Internal Medicine, Cleveland Clinic Foundation, Cleveland, Ohio USA; Jacobi Medical Center, North Central Bronx Hospital, New York, NY USA; Columbia University Medical Center, New York, NY USA; Khyber Medical College, Peshawar, Pakistan

## Abstract

**Background:**

Gastrointestinal stromal tumor (GIST) is a rare tumor comprising 0.1-0.3% of all gastrointestinal (GI) malignancies. Stomach followed by small intestine is the most common sites of involvement, implicated in 95% of the cases. We present a case of GIST complicating a colonic interposition. To the best of the author's knowledge, this is the first reported case of GIST complicating a colonic interposition.

**Case presentation:**

A 47 year old African American male presented to the emergency department with intermittent, severe chest pain. Past medical history was significant for alkali (NaOH) ingestion during 1980 for which esophageal resection and a colonic pull-through was performed. A CXR revealed a widened mediastinum and CT scan chest revealed showed a large (11.4 × 8.3 × 12.1 cm) vascular mediastinal mass. At endoscopy, a large, ulcerated, cratered and friable mass was found at 29cm extending to 36cm at which point the lower anastomosis of the colonic pull through was present. Multiple endoscopic biopsies were obtained which showed that the tumor was immunoreactive with CD117, CD34 and DOG1 while markers of carcinoma, melanoma and lymphoma were negative. In light of the pathology report, the immunohistochemistry and the CT scans, the tumor was classified as a stage 4 GIST of colonic interposition.

**Conclusions:**

GIST can complicate unusual locations such as colonic interposition and should be kept in the differential diagnosis of such unusual presentations.

## Background

Gastrointestinal stromal tumor (GIST) is a rare tumor comprising 0.1-0.3% of all gastrointestinal (GI) malignancies. The most common location of GIST is the stomach (60%), followed by the small intestine (35%). On rare occasions, these tumors can also be found in other regions of the gastrointestinal tract (GIT). Less than 5% of these tumors originate from the colon, making colonic GIST extremely rare [1, 2]. Despite the broad range of clinical presentations, many cases are asymptomatic, making early diagnosis difficult.

Our patient underwent colonic interposition for the treatment of caustic ingestion and developed a GIST in this surgical site.

## Case presentation

A 47 year old African American male presented to the emergency department with intermittent, severe chest pain associated with palpitations and one episode of vomiting.. The pain started without any preceding event and persisted for approximately 30 seconds during both rest and exercise. The patient claimed to use street cocaine for the treatment of his pain. During the last few months the patient experienced a 40 pound weight loss. The patient had no history of cardiac symptoms and had a good exercise tolerance. Patient’s past medical history was significant for alkali (NaOH) ingestion during 1980 for which esophageal resection and a colonic pull-through was performed. Notable aspects of the patient’s history included a father with a brain tumor, 20 pack year history of smoking and a regular consumption of cocaine for chest pain.

On physical examination, the patient was alert, cooperative and in slight distress. His vitals were normal except for a blood pressure of 147/83. General examination showed a few slightly enlarged cervical lymph nodes bilaterally. Examination of the chest revealed a 4 × 4 cm midline mass which was extremely tender and inspection of abdomen showed an old surgical scar.

In the emergency room the patient received aspirin 325 mg, morphine 4 mg intravenously and famotidine 20 mg intravenously.

**Figure 1:**
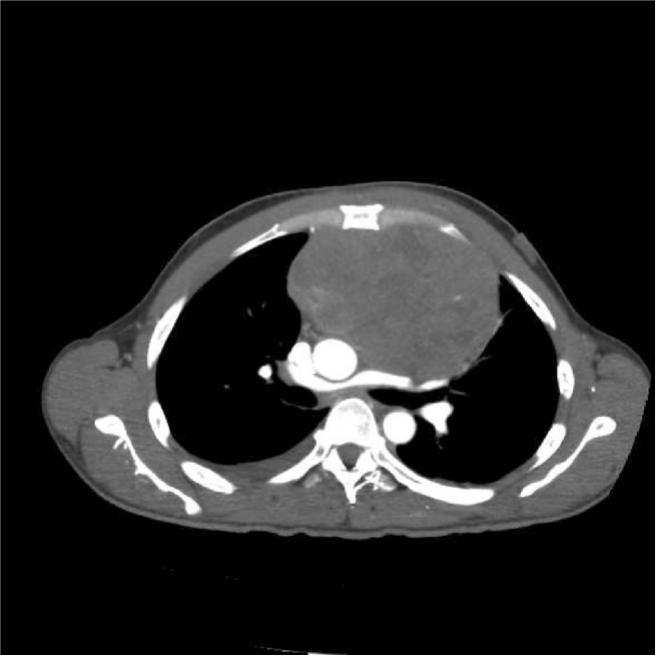
**Chest CT scan showing a mediastinal mass.**

Baseline laboratory tests including complete blood count, metabolic and coagulation profiles were within normal limits. An unremarkable electrocardiogram (EKG) and three sets of negative troponins excluded a cardiac cause for patient’s chest pain. A Chest X Ray (CXR) revealed a widened mediastinum and a CT Chest was ordered which was eventually extended to include the abdomen and pelvis. This showed a large (11.4 × 8.3 × 12.1 cm) vascular mediastinal mass (Figure 1).

**Figure 2:**
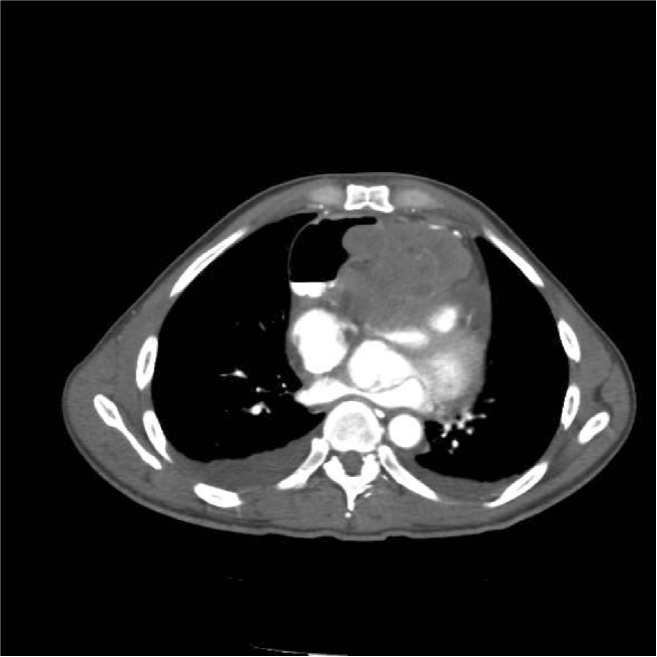
**CT scan showing proximity of the mass with the great vessels.**

This mass was in contiguity with the heart, stomach and aorta, exerting mass effect on the aorta and pulmonary vasculature (Figure 2).

**Figure 3:**
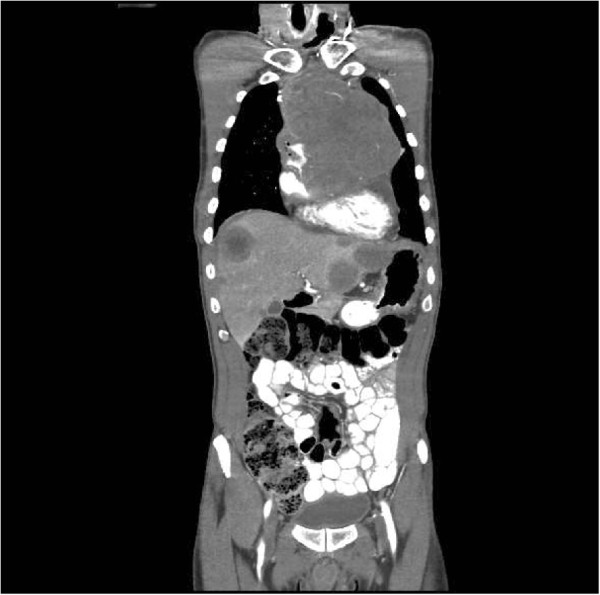
**CT scan abdomen showing multiple enhancing hepatic lesions.**

The CT scan also revealed multiple round enhancing liver lesions raising the suspicion of a metastatic malignancy with the largest lesion in the left lobe measuring 3.9 cm (Figure 3).

**Figure 4:**
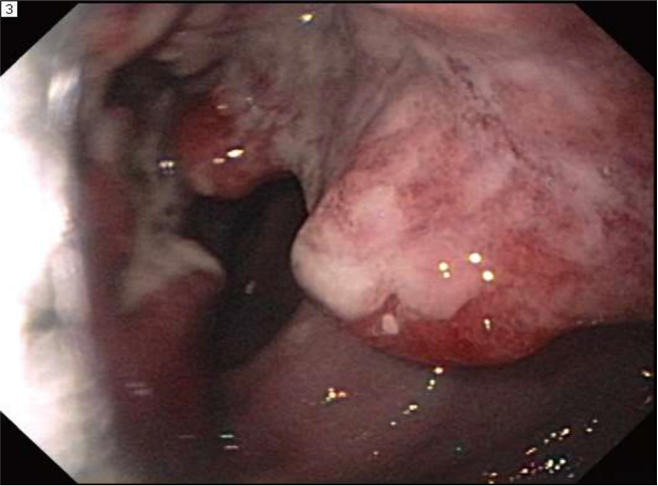
**Endoscopic visualization of an ulcerated, friable mass.**

GI and Surgery were consulted and an endoscopy and EUS were planned. At endoscopy, a large, ulcerated, cratered and friable mass was found at 29 cm extending to 36 cm at which point the lower anastomosis of the colonic pull through was present (Figure 4).

**Figure 5:**
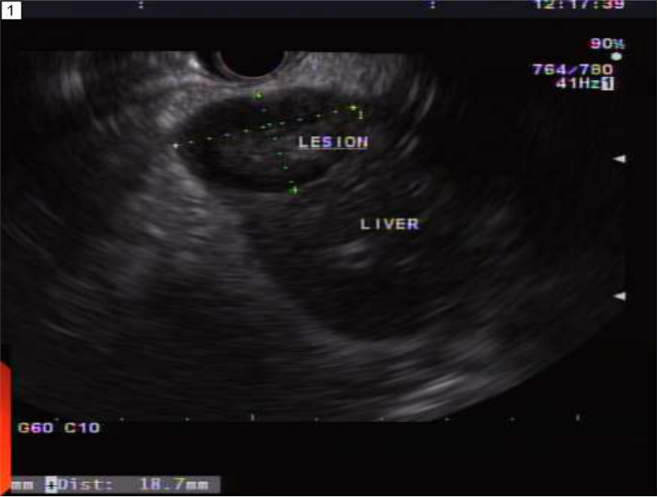
**Ultrasound appearance of hepatic lesions.**

Multiple endoscopic biopsies were obtained and sent for pathology. Fine needle aspiration was attempted under EUS guidance to assess the nature of hepatic lesions (Figure 5).

**Figure 6:**
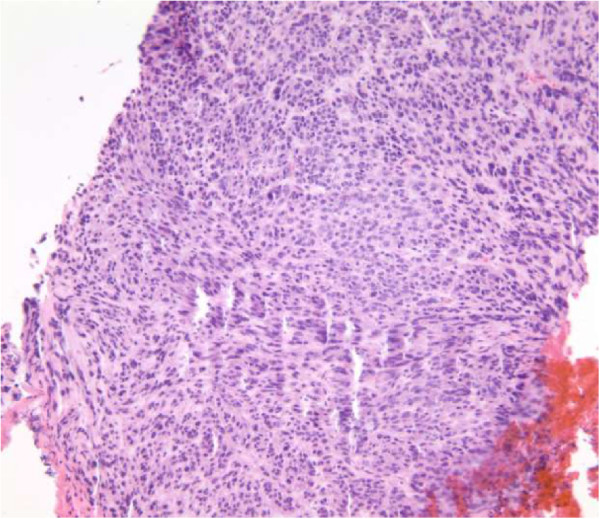
**H and E stained colonic mucosa.**

**Figure 7:**
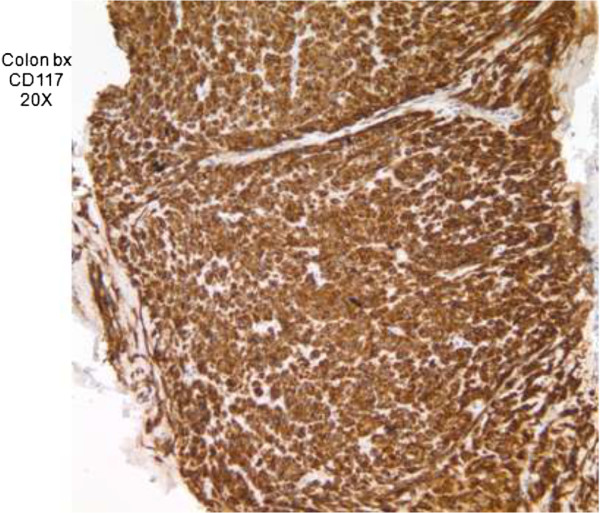
**CD117 stained colonic mucosa.**

**Figure 8:**
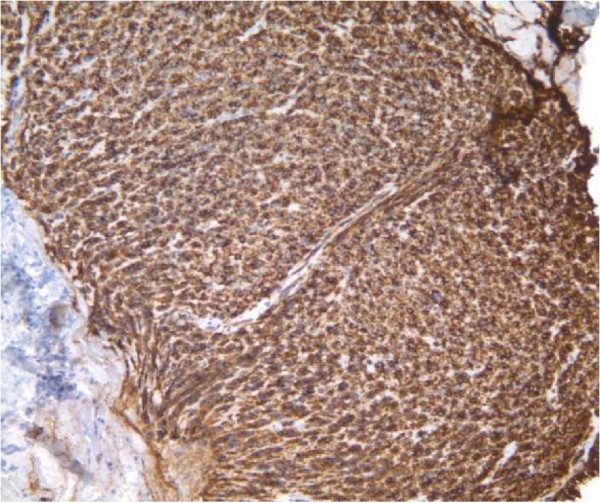
**CD117 stained colonic mucosa.**

Pathology of the esophago-gastroduodenoscopy (EGD) biopsy revealed colonic mucosa with acute and chronic inflammation, granulation tissue and ulcerative debris. The fragments were highly atypical with hyperchromasia and mitotic activity. The FNA from hepatic lesions were also positive for malignant cells. The immunohistochemical analysis of the EGD biopsy showed that the tumor was immunoreactive with CD117, CD34 and DOG1 while markers of carcinoma, melanoma and lymphoma were negative. In light of the pathology report, the immunohistochemistry and the CT scans, the tumor was classified as a stage 4 GIST of colonic interposition (Figures 6, 7 and 8).

Subsequent to the biopsy based diagnosis, surgical and radiological treatment modalities were ruled out due to the extent and nature of the tumor. The patient was administered imatinib 400 orally daily, adequate pain control medications and a suitable bowel regimen.

**Figure 9:**
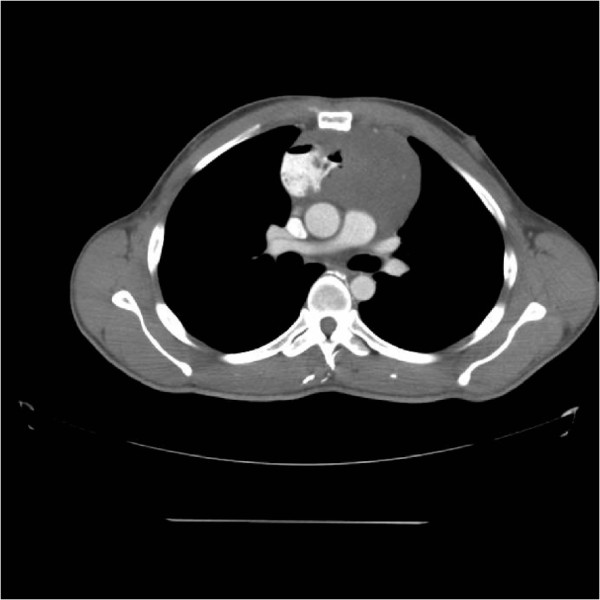
**CT scan showing resolution of tumor at 3 months.**

**Figure 10:**
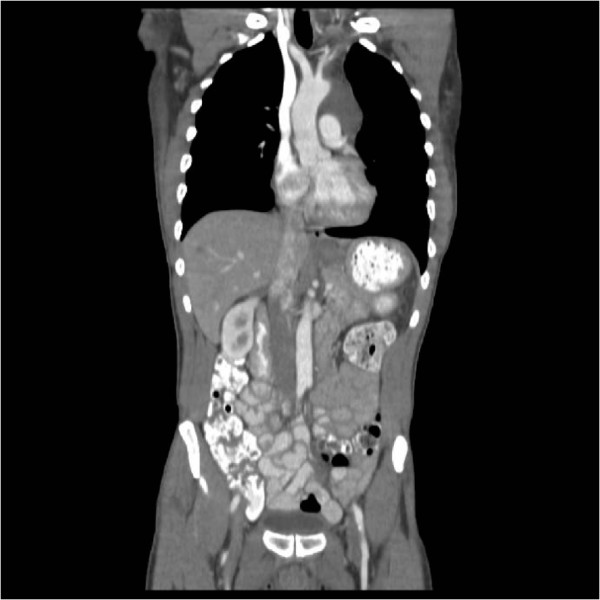
**CT scan showing resolution of tumor at 3 months.**

Patient continues to do well on 3 months follow up. Follow CT scan shows considerable shrinkage in the size of the tumor Figures 9 and 10.

## Discussion

The present case apprears to be unique. Despite an extensive literature search, no previous cases of GIST complicating a colonic interposition have been reported.

Retrosternal coloplasty remains the gold standard for the management of esophageal reconstruction secondary to caustic ingestion [3]. Primarily used in the treatment of esophageal cancer, interposition grafts are routinely performed. Multiple studies indicate that over 25% of patients undergoing this surgical procedure develop some type of complication, including anastomotic leakage, stenosis, wound infection, intestinal obstruction and aspiration pneumonia, among others [4]. Neoplastic lesions complicating a colonic interposition are exceedingly rare. As of 2010, just 10 such cases were reported in the literature. The majority of lesions were adenocarcinomas or tubular adenomas [5].

The patient in this report developed a GIST almost 2 decades after the procedure was performed, closely following similar cases in the literature where the interposition was complicated by a neoplastic lesion after a decade or more. It is generally believed that patients with esophageal malignancy have an already dismal prognosis and hence with a reduced life span, the chances to develop another primary malignancy in the interposition are fairly low.

As the most common mesenchymal neoplasm of the GI tract, GIST manifests itself inconsistently in patients and requires immunohistochemical means to establish a definitive diagnosis. Studies indicate that approximately 95% of the cases are KIT positive which confers the tumor with proliferative potential as well as the ability to evade apoptotic pathways [6].

The radiographic features of a colonic GIST mimic those of leiomyosarcomas, appearing as transmural or sub-mucosal masses on barium follow through. The differential diagnosis also includes adenocarcinoma, lymphoma, metastatic melanoma and retroperitoneal sarcoma [7]. A case series of colonic GIST demonstrated that roughly half the patients with lesions greater than 1 cm, showed evidence of metastasis while one fifth had hepatic metastastases as evident in the present patient [7].

The predominant histopathological forms of the tumor are spindle cell and epitheloid. CD117 (KIT) is the primary immunological marker, seen in roughly 75% of cases of colonic GIST (compared with 95% of cases of gastric origin). Other less frequent immunological markers include CD34 and alpha smooth muscle actin [8].

Imatinib mesylate, an inhibitor of the tyrosine kinase signaling pathway is the most effective treatment option. The disease carries a dismal prognosis in the presence of overt metastasis. However, the subject patient showed a dramatic response to imatinib therapy and is doing well after a three month follow up. Consensus Tumor size and the presence of numerous mitosis are the most important determinants affecting prognosis [8].

## Conclusions

GIST can complicate unusual locations including the colonic interposition and should be included in the differential diagnosis of such unusual presentations.

## Consent

Written informed consent was obtained from the patient for publication of this case report and any accompanying images. A copy of the written consent is available for review by the Editor-in-Chief of this journal.
